# Repurposing drugs against Alzheimer’s disease: can the anti-multiple sclerosis drug fingolimod (FTY720) effectively tackle inflammation processes in AD?

**DOI:** 10.1007/s00702-023-02618-5

**Published:** 2023-04-04

**Authors:** Volkmar Leßmann, Georgia-Ioanna Kartalou, Thomas Endres, Marc Pawlitzki, Kurt Gottmann

**Affiliations:** 1 Institute for Physiology, Medical Faculty, Otto-Von-Guericke-University, Leipziger Str. 44, 39120 Magdeburg, Germany; 2 Center for Behavioral Brain Sciences, Magdeburg, Germany; 3 Institute of Neuro- and Sensory Physiology, Medical Faculty, Heinrich Heine University, Duesseldorf, Germany; 4 Department of Neurology, Medical Faculty and University Hospital Düsseldorf, Duesseldorf, Germany

**Keywords:** Alzheimer’s disease, Dementia, Drug repurposing, fingolimod, FTY720, APP/PS1, Spines, LTP, Spatial memory, Microglia, Astrogliosis, Neuroinflammation, BDNF, Hippocampus, Learning and memory, Neurodegenerative disease, Sphingosine-1-phosphate receptor, Ozanimod, Siponimod, Lecanemab, Gantenerumab, Aducanumab

## Abstract

Therapeutic approaches providing effective medication for Alzheimer’s disease (AD) patients after disease onset are urgently needed. Previous studies in AD mouse models and in humans suggested that physical exercise or changed lifestyle can delay AD-related synaptic and memory dysfunctions when treatment started in juvenile animals or in elderly humans before onset of disease symptoms. However, a pharmacological treatment that can reverse memory deficits in AD patients was thus far not identified. Importantly, AD disease-related dysfunctions have increasingly been associated with neuro-inflammatory mechanisms and searching for anti-inflammatory medication to treat AD seems promising. Like for other diseases, repurposing of FDA-approved drugs for treatment of AD is an ideally suited strategy to reduce the time to bring such medication into clinical practice. Of note, the sphingosine-1-phosphate analogue fingolimod (FTY720) was FDA-approved in 2010 for treatment of multiple sclerosis patients. It binds to the five different isoforms of Sphingosine-1-phosphate receptors (S1PRs) that are widely distributed across human organs. Interestingly, recent studies in five different mouse models of AD suggest that FTY720 treatment, even when starting after onset of AD symptoms, can reverse synaptic deficits and memory dysfunction in these AD mouse models. Furthermore, a very recent multi-omics study identified mutations in the sphingosine/ceramide pathway as a risk factor for sporadic AD, suggesting S1PRs as promising drug target in AD patients. Therefore, progressing with FDA-approved S1PR modulators into human clinical trials might pave the way for these potential disease modifying anti-AD drugs.

## Introduction

Dementia is one of the greatest global challenges for health and social care in the twenty-first century. It occurs mainly in people older than 65 years, so increases in numbers and costs are driven, worldwide, by increased longevity in most societies, resulting from the welcome reduction in people dying prematurely (Livingston et al. 2017). Globally, ~ 50 million people are currently living with dementia, and this number is projected to triple by 2050. The 2015 global costs of dementia were > 800 billion € per year, and this figure will continue to increase as the number of people with dementia rises (Livingston et al. 2017).

Alzheimer’s disease (AD) is the most common (~ 60%) form of dementia resulting in a progressive decline of cognitive functions. AD is characterized by formation of amyloid plaques and neurofibrillary tangles in the brain, which initiate a multi-facetted array of cellular, synaptic, inflammatory, and immunological responses that—together—trigger progressive loss of memory storage and memory retrieval in neuronal circuits. Amyloid-β (Aβ) peptides and tau proteins are the primary components of amyloid plaques and neurofibrillary tangles that are characteristic for AD (Congdon and Sigurdsson 2018).

It is generally agreed on that by the time when AD symptoms become detectable, a combination of different pathologies in the brain, which can be traced back to different molecular and cellular processes, contributed to this process: (1) Aβ protein accumulations and deposits (plaques) in the cerebral cortex (neocortex), which begin ~ 10–15 years before the onset of AD symptoms. (2) Accumulation of pathologically changed (i.e., strongly phosphorylated) variants of the microtubule associated tau protein starting in transentorhinal and hippocampal neurons, which begins a few years before the onset of AD symptoms (Goedert and Spillantini 2006). (3) Neuron-damaging inflammatory processes of the brain, which are triggered by the brain's immune cells (microglia) and astrocytes that under healthy physiological conditions support the function of neurons. These inflammatory processes are a reaction of the brain to the Aβ and tau pathology that deteriorate with ongoing disease progression. This sequence of events supports the view that AD pathogenesis is not restricted to neurons, but involves immunological mechanisms in the brain. Misfolded and aggregated proteins bind to receptors on microglia and astroglia, and trigger an innate immune response, characterized by release of inflammatory cytokines and other immunological modulators that promote AD progression. In this respect, genome-wide omics analysis identified candidate genes that increase the risk for sporadic AD, which encode proteins that regulate (microglial and astroglial) clearance of misfolded proteins and inflammatory responses. This list of risk factors includes, e.g., the ABC transporter A7 that is involved in clearance of Amyloid-β from the cerebrospinal fluid (CSF), Apolipoprotein E that regulates lipid metabolism, and TREM2 that is involved in microglia mediated clearance of tau. Of note, two thirds of these risk genes are strongly expressed in microglia, stressing their potential role as promising targets for AD therapy (Jansen et al. 2019; McQuade and Blurton-Jones 2019). On the same vein, systemic inflammation and obesity are likely to interfere with immunological processes in the brain, thereby promoting AD progression (Heneka et al. 2015).

While lifestyle factors such as physical exercise or a healthy diet might be able to delay the disease onset, more than 30 years of dementia research have not yet led to a disease modifying therapy in human AD patients. Thus, currently approved AD medications consist of symptomatic treatments such as acetylcholine esterase inhibitors (donepezil, rivastigmine, and galantamine), NMDA-R antagonists (memantine), and a combination therapy thereof (memantine/donepezil). In 2021, the amyloid-lowering monoclonal antibody Aducanumab was debatably approved as an anti-AD causal therapy although it could not reliably slow cognitive decline in AD patients. Similar negative results were obtained in 2022 for Gantenerumab (Bateman et al. 2022), whereas very recently, Lecanemab was the first Aβ monoclonal antibody that was shown to slightly delay cognition deficits in early AD patients (van Dyck et al. 2023). While the latter study supported a causal connection between Aβ plaque load and decreased cognitive performance in AD patients, it remains to be shown whether Lecanemab can also mitigate clinical meaningful cognitive decline, especially at later disease stages. Thus, there is still an unmet need for AD treatments that can improve brain function and cognition. The failure of most of the Aβ antibody-based clinical trials in recent years to improve cognition in AD patients is likely due to the fact that in advanced stages of AD, inflammation-related processes have already started and are responsible for the rapid increase in neurodegeneration. At this stage of AD, even with an effective reduction in Aβ, the subsequently induced inflammation has reached a status where Aβ reduction alone cannot stop AD progression [compare also (Heneka et al. 2015)].

Accordingly, therapeutic interventions focusing on reduction in neuro-inflammatory signaling are promising approaches to ameliorate AD-related deficits. Therefore, repurposing federal drug administration (FDA) approved anti-inflammatory drugs with well-established preclinical mechanisms of action on AD pathology offer an important opportunity for clinical translation and human AD trials.

## Repurposing of FDA-approved drugs for AD: focus on fingolimod

### Why is fingolimod an appropriate candidate?

Repurposing pharmaceutical therapies already developed for one disease that could help to treat another can be a faster, cheaper and safer way to move into the clinic. Many attempts to develop disease modifying therapies against AD have focused on reducing Aβ and tau pathologies. However, additional inflammatory processes in the brain of AD patients were obvious already when Alois Alzheimer discovered the disease (Alzheimer 1907). In the past ten years, the evidence for an involvement of microglia and astrocytes in the inflammatory processes accompanying AD pathology became overwhelming [see, e.g., (Heneka et al. 2015)]—although these processes are not specific for AD, but rather occur in other neurodegenerative diseases alike [see, e.g., (Salter and Stevens 2017)]. Since certain subtypes of Multiple sclerosis (MS)—another immunological/inflammatory disease of the brain—can be treated since 2011 quite successfully with the sphingosine-1-phosphate receptor modulator Fingolimod (FTY720, Gilenya), it seems reasonable to study its effects in other neurodegenerative diseases that involve inflammation.

Fingolimod is an approved (FDA and European Medicines Agency (EMA)) drug for the treatment of relapsing, remitting multiple sclerosis (RRMS) since 2011, and is widely prescribed in clinical practice (Druart et al. 2018). Accordingly, fingolimod targets, cellular receptors, therapeutic properties, and potential side effects are known in all details. While patients have to be monitored for cardiac functions upon first medication with fingolimod, patients not showing this effect upon first medication have never been reported to develop cardiological symptoms later during continued medication. The cardiological side effects are most likely due to actions of fingolimod receptors in heart and vasculature. Prolonged fingolimod treatment can lead to lymphopenia and therefore can be contraindicated in immune suppressed patients or patients suffering bacterial/viral infectious diseases [compare (Druart et al. 2018)]. Of note, the immunosuppressive (side-) effects of FTY720 are reversible and discontinued medication can cure these adverse properties.

### Fingolimod receptors and downstream signaling pathways

Fingolimod has emerged recently as a promising neuroprotective agent in a wide range of CNS diseases, including Alzheimer’s disease (AD; reviewed, e.g., in Angelopoulou and Piperi 2019; Bascunana et al. 2020; Pournajaf et al. 2022). Importantly, fingolimod tackles those immunological and neuro-inflammatory processes that are triggered by amyloid-β and tau fibrillary tangle pathology (Aytan et al. 2016; Baloni et al. 2022; Bascunana et al. 2023; Fagan et al. 2022; Kartalou et al. 2020).

Fingolimod is a structural analog of sphingosine and oral FTY720 application to human Multiple sclerosis (MS) patients is sufficient for effective uptake into the blood stream. Importantly, fingolimod readily penetrates the CNS of rodents (Foster et al. 2007) and humans (Briard et al. 2011). In target tissue, it can be converted by sphingosine kinase 2 (SphK2) to fingolimod-phosphate, which is a potent modulator of the five homologous G-protein coupled sphingosine-1-phosphate receptors (S1PR1–5; (Angelopoulou and Piperi 2019)). In the immune system, fingolimod-induced S1PR modulation promotes the retention of T cells in lymph nodes, thereby reducing the invasion of the CNS by auto-reactive lymphocytes, where they would otherwise promote demyelination (Chaudhry et al. 2017). S1PRs are found on numerous hematopoietic and immune cells, including microglia, but are also expressed in many other organs, including heart and vasculature (Chaudhry et al. 2017). More recent studies suggest an additional role of fingolimod in suppressing tau-phosphorylation (Wang et al. 2021; Yin et al. 2021), which represents another hallmark of AD pathology in human patients.

Several studies reported neuroprotective effects of fingolimod on Aβ pathology, APP metabolism, AD-related memory deficits, BDNF elevation, and neuro-inflammation and were nicely summarized in recent reviews (compare Angelopoulou et al. 2019; Bascunana et al. 2020; Pournajaf et al. 2022). There is strong evidence that fingolimod may contribute to the inhibition of Aβ accumulation by reducing the Aβ load in the brain (Aytan et al. 2016; Carreras et al. 2019; Kartalou et al. 2020; McManus et al. 2017; Takasugi et al. 2013). Several mechanisms associated with this hypothesis have been proposed including the blockage of Aβ production in neurons via suppression of APP processing by γ-secretase involving S1P1 receptors and the G_i_ pathway (Lee et al. 1996). Moreover, it has been suggested that fingolimod by antagonizing endogenous S1P acts in a protective way in the regulation of β-secretase activity and thereby reduces generation of Aβ (Takasugi et al. 2013). Also, the beneficial role of fingolimod and specifically fingolimod-P in reducing Aβ is strengthened by the simultaneous correlation of SphK2 levels (reviewed in Angelopoulou et al. 2019).

Previous studies described the neuroprotective role of fingolimod in different diseases and numerous molecular mechanisms have been suggested to underlie these effects (Angelopoulou et al. 2019). It has been shown that fingolimod protects from neuronal death by blocking the neurotoxic effects of oligomeric Aβ (Asle-Rousta et al. 2013; Doi et al. 2013; Hemmati et al. 2013; Ruiz et al. 2014). Specifically, there is evidence that suppressing the activation of caspase-3 is associated with the inhibition of neuronal apoptosis that is induced by the toxic properties of Aβ (Asle-Rousta et al. 2013; Hemmati et al. 2013). Interestingly, fingolimod was shown to mitigate learning deficits in mice injected with Aβ to mimic AD-like amyloidosis, and this beneficial effect was mediated by increased expression of brain-derived neurotrophic factor (BDNF) and downstream tropomyosin related kinase (TrkB) receptor signaling (Doi et al. 2013; Fukumoto et al. 2014). Other studies provided evidence that fingolimod is involved in the reduction of toxic glutamate levels and that this effect is mediated by the S1P receptor, thereby protecting neuronal loss mediated by NMDA receptor-dependent excitotoxicity (see, e.g., Aytan et al. 2016; Di Menna et al. 2013), reviewed in (Angelopoulou and Piperi 2019).

There is evidence that fingolimod counteracts AD-associated neuro-inflammation by regulating the activation of microglia and astrocytes as well as their pro-inflammatory mediators (Aytan et al. 2016; Kartalou et al. 2020; Mirzaei et al. 2022; Rothhammer et al. 2017; Zhong et al. 2019). Specifically, it is suggested that fingolimod treatment reduces microgliosis and astrogliosis. On the same vein, fingolimod was proposed to promote the increased production of selective signal transducer and activator of transcription 3 (STAT3), which is a crucial player in the transformation of the M1 pro-inflammatory microglial phenotype to the M2 anti-inflammatory phenotype [(Qin et al. 2017), compare (Paolicelli et al. 2022)]. Regarding the effect of FTY720 on astrocytes in AD it has been reported that it reduces astrocytosis and inhibits the secretion of pro-inflammatory molecules from astrocytes. Moreover, there is evidence that fingolimod induces the generation of several neurotrophic factors in astrocytes, and that it is associated with reduced expression of several TNF induced inflammatory cytokines (Hoffmann et al. 2015; Janssen et al. 2015).

It is very encouraging that fingolimod has been demonstrated to exert beneficial effects also on cognitive decline in AD mice (Aytan et al. 2016; Baloni et al. 2022; Fagan et al. 2022; Kartalou et al. 2020). Specifically, there are study results suggesting that treatment with this drug can improve Aβ-associated impaired synaptic function and ameliorate memory deficits, thereby restoring cognitive functions. Altogether, these findings suggest that fingolimod and related S1PR modulators might have beneficial clinical effects in patients suffering from AD.

### Evidence for beneficial effects of fingolimod in rodent models of AD

In recent years, several studies suggested that fingolimod protects neurons from Aβ-toxicity both in vitro (Doi et al. 2013; Ruiz et al. 2014) and in vivo (Asle-Rousta et al. 2013; Fukumoto et al. 2014; Hemmati et al. 2013), but its cellular mode of action remained unclear. Given the rich expression of S1PRs in microglia, which are intimately connected with Aβ plaques and actually cover the plaque surface, fingolimod might regulate Aβ dissolution from plaques and oppose microglia mediated inflammatory mechanisms (Heneka et al. 2015; Sarlus and Heneka 2017), occurring in advanced stages of AD. Recent reports, showing an FTY720 induced up-regulation of neuronal brain-derived neurotrophic factor (BDNF) expression (Deogracias et al. 2012; Doi et al. 2013; Fukumoto et al. 2014) and rescue of synaptic NMDA receptors in Aβ-treated neurons (Joshi et al. 2017), indicate synapto-protective effects of fingolimod in different AD models in vitro. Since synaptic dysfunction is one of the earliest features of AD (Mucke and Selkoe 2012; Selkoe 2002), fingolimod may, thus, prevent detrimental neuro-inflammatory secondary effects when applied early on in the disease.

In 2016, Aytan and colleagues (2016) showed that oral fingolimod treatment starting already at 4 weeks of age (and continued lifelong) in 5xFAD mice (an AD mouse model harboring 5 mutated proteins associated with human familial AD) prevented development of Aβ plaque pathology, microgliosis, and astrogliosis. The same lifelong treatment was suggested later to also reduce in a dose-dependent manner deficits in hippocampus-dependent Morris water maze (MWM) learning (Carreras et al. 2019). These studies shed light on a possible *protective* role of fingolimod to hinder development of AD pathology.

In 2020, fingolimod (i.p. injection) was described for the first time to rescue AD pathology, synaptic dysfunction and associated memory deficits when treatment started after onset of disease pathology (i.e., at 5–6 months of age) and was continued for only 4 weeks (Kartalou et al. 2020). These experiments were performed in a different amyloid precursor protein/presenilin-1 (APP/PS1) AD mouse model (Radde et al. 2006) than that used in previous studies (Aytan et al. 2016; Carreras et al. 2019), and completely rescued synaptic deficits (i.e., reduced number of synaptic spines and reduced long-term potentiation in hippocampal CA1 pyramidal neurons) that were present in sham-treated APP/PS1 animals (Kartalou et al. 2020). Importantly, such a medication starting no earlier than after onset of memory dysfunctions would—of course—be highly desirable for treating human patients only when they have just been diagnosed for cognitive impairments that are likely to be caused by onset of AD. In this respect, the currently ongoing strong initiatives to diagnose the onset of AD, based on fluid blood and cerebrospinal fluid (CSF) biomarkers, or by MRI and PET neuroimaging of hippocampal and cortical brain structures in human patients [reviewed, e.g., in (Agarwal et al. 2021; Weiner et al. 2022)] could be ideally complemented by a disease modifying FTY720 anti-neuroinflammatory therapy.

The rescuing effect of fingolimod—when treatment started briefly after onset of disease symptoms—on synaptic and memory dysfunction that is accompanied by reduced microgliosis, astrogliosis and mildly reduced Aβ pathology (Kartalou et al. 2020) has meanwhile been confirmed independently by other labs, employing either a distinct APP/PS1 AD mouse model (Baloni et al. 2022), or in a combined amyloidogenic/tauopathy AD mouse model (Fagan et al. 2022). Together, these studies provide a strong case for fingolimod effectively rescuing AD symptoms and pathology, independent of the specific AD mouse model used. The accompanying reduction in microgliosis and astrogliosis, which has been observed in all above mentioned studies suggests that anti-inflammatory mechanisms are involved in this effect. Unexpectedly, while enhanced BDNF/TrkB signaling mechanisms have previously been described to underlie disease ameliorating fingolimod actions, e.g., in mouse models of Rett syndrome and Huntington’s disease (Deogracias et al. 2012; Di Pardo et al. 2014; Miguez et al. 2015), such BDNF/TrkB mechanisms have not been observed in AD mice (Kartalou et al. 2020).

### The cellular sphingosine/ceramide pathway that generates S1P is a drug target in human sporadic AD

Of utmost importance, using a multi-omics approach in a genome-wide association study in roughly 2000 patients, Baloni and colleagues (2022) provided strong evidence that mutations in genes encoding enzymes of the sphingosine/ceramide (SM) pathway come with an increased risk of being diagnosed for sporadic AD in human patients. More specifically, the authors identified genetic variants in seven of the 35 genes in the SM pathway to be significantly associated with AD and its (bio)markers (Baloni et al. 2022), Supplementary Table 3), which covered the whole spectrum of Amyloid-β, Tau, Neurodegeneration, and Cognition (A–T–N–C) measures of AD (Jack et al. 2018).

Of note, reaction products of the sphingosine/ceramide cell membrane lipid pathway are upstream to S1P receptor (S1PR) signaling. Consequently, these results clearly identify the sphingosine/ceramide pathway as a drug target for AD treatment—with fingolimod and structurally related compounds already at hand (siponimod, ozanimod, ponesimod all approved for relapsing or progressive MS by FDA and EMA) to treat AD in humans.

### Is there a specific microglia subtype that rules AD pathology and is susceptible to fingolimod regulation?

Fingolimod treatment in different AD mouse models (compare previous section) decreased microglia activity compared to untreated AD mice, as probed by Iba1 immunoreactivity, reduced astrogliosis, as probed with glial fibrillary acidic protein (GFAP) immunohistochemistry (Carreras et al. 2019; Kartalou et al. 2020), as well as decreased the number of infiltrating lymphocytes (Aytan et al. 2016; Fagan et al. 2022). Interestingly, it has been reported that reactive microglia are the primary sensors of pathology in AD (as in other neurodegenerative disorders like Huntington’s disease (HD), Amyotrophic lateral sclerosis (ALS), and Parkinson’s disease (PD). Amyloid-β and fibrillary tangle-associated proteins are detected by resting microglial cells, converting them to reactive M1 microglia [compare (Paolicelli et al. 2022)]. These microglia then release Il-1β, TNF, and complement component 1, subcomponent q (C1q), thereby transforming resting astroglia to A1 reactive astrocytes with elevated expression of complement factor C3 (Liddelow and Sofroniew 2019). This reactive subpopulation of astroglia then induces synaptic degeneration and neuronal loss through release of toxic phospholipids and proteins, as well as additional complement factors that attract lymphocytes, followed by liberation of fragmented mitochondria from astrocytes and microglia that propagate the neuro-inflammation (Barbar et al. 2020; Habib et al. 2020; Joshi et al. 2019; Liddelow et al. 2017). Interestingly, A1 reactive astrocytes can make up a relatively small fraction of the overall astroglia in diseased tissue, thereby escaping detection by conventional markers of activated astrocytes, like, e.g., GFAP. Detection of A1 reactive astrocytes, therefore, often requires single-cell transcriptome analysis. Given that fingolimod is well established to decrease lymphocyte infiltration in the CNS of MS patients, the beneficial actions of fingolimod in AD mouse models might be related to egress of lymphocytes from the brain (Liddelow et al. 2017). Thus, searching the cellular target(s) for the beneficial fingolimod actions in AD mice will require comprehensive analysis of microglia, astrocytes, and lymphocytes.

### Potential use of fingolimod-related S1PR modulators to treat AD in humans

Fingolimod binds with comparable affinity to all 5 subtypes of S1PRs (i.e., S1P1 through S1P5). The improved understanding of fingolimod's mode of action and the role of S1PR subtypes has provided strategies for the development of the more selective second-generation S1PR modulators. With regard to potential side effects of fingolimod, a more selective S1PR modulation serves to reduce the most important safety concerns regarding cardiac-related side effects (Roy et al. 2021). Against the background of no requirement for phosphorylation for activation (as required for fingolimod) and the shorter half-lives of these second-generation agents, the importance of fingolimod in MS treatment landscape has already started to decrease. Of note, all S1P1 modulators inhibit lymphocyte egress out of secondary lymphoid organs, resulting in a profound diminution of naive and central memory T cells and memory B cells in the periphery (Comi et al. 2017). The resulting peripheral lymphopenia increases the risk of infections and seems to reduce humoral and cellular vaccination responses, which should be considered (Meyer-Arndt et al. 2022).

Siponimod (BAF312, Mayzent) was the first new-generation sphingosine-1-phosphate receptor (S1PR) modulator, which was already approved for relapsing and progressive MS by FDA and for active secondary progressive MS by EMA. Siponimod shows a high affinity at two of five S1PRs, namely S1P1 and S1P5 and does not require phosphorylation. In the EXPAND study, siponimod met its primary endpoint of reduced risk of 3-month confirmed disability progression. Interestingly, in this phase III trial, the frequency of adverse events (AE) was just slightly higher in siponimod-treated patients (89%) than in the placebo group (82%). Similar results were also observed for serious AE [SAE; 15% vs. 18%; (Kappos et al. 2018)]. Moreover, recent real-world data as well as results from the open-label extension phase of the EXPAND trial suggest sustained clinical achievement over 5 treatment years including stable cognitive performance (Benedict et al. 2021).

Like siponimod, ozanimod (Zeposia) no longer needs to be phosphorylated for activation. Ozanimod and its main metabolites bind in vivo to the S1PRs S1P1 and S1P5, with a tenfold preference for S1P1. In contrast, ozanimod does not show significant binding to S1P2, S1P3, and S1P4 receptors. Ozanimod exerts its main effect through functional antagonism at S1P1, whereas it is still unclear whether ozanimod exerts a relevant effect on brain parenchymal cells via S1P5. In the phase III SUNBEAM study, ozanimod was tested in relapsing MS patients and compared with interferon beta-1a (30 μg). As expected, ozanimod significantly reduced relapse rate compared with interferon beta-1a treated MS patients. Notably, no first-dose, clinically significant bradycardia or further cardiac side effects were reported (Comi et al. 2019). Overall, ozanimod is licensed for RRMS by the EMA and for clinically isolated syndrome, RRMS, and active SPMS (i.e., Secondary Progressive Multiple Sclerosis) by the FDA. Moreover, ozanimod showed beneficial effects in patients with ulcerative colitis (Sandborn et al. 2021).

Ponesimod (Ponvory) is another active metabolite and S1PR modulator of the second generation that was approved for treatment of MS (EMA: active RMS; FDA: clinically isolated syndrome, RRMS, and active SPMS). In the OPTIMUM trial, ponesimod was tested against teriflunomide and met the primary endpoint of annual relapsing rate (Kappos et al. 2021). Similarly to ozanimod, cardiac side effects were not common. However, one explanation for reduced cardiac side effects may be related to an altered titration schedule of both ozanimod and ponesimod compared with fingolimod. Of note, further next-generation S1PR modulators are currently being developed for MS and/or inflammatory bowel diseases including amiselimod, ceralifimod, and etrasimod (Peyrin-Biroulet et al. 2017).

In conclusion, it remains to be investigated whether the above mentioned S1PR modulators that have recently been approved for MS and ulcerative colitis will show similar anti-neuroinflammatory effects in AD mouse models as fingolimod. If yes, especially ozanimod and siponimod—because of absence of cardiac side effects— might be even better suited to become evaluated for treating AD patients.

### Considerations regarding the use of fingolimod and related compounds in human clinical trials on AD

In the light of the very promising preclinical results in AD mouse models, where temporary FTY720 treatment starting after onset of AD symptoms rescues synaptic and memory deficits (compare “Evidence for beneficial effects of Fingolimod in rodent models of AD”), and given the battery of structurally related (second-generation) S1PR modulators that are already FDA and EMA approved for treating different inflammatory diseases in humans (compare previous paragraph), it seems logical to assess their potential for the treatment of AD in human clinical trials.

In accordance with previous clinical trials designed to assess the disease modifying potential of drugs against AD [compare (van Dyck et al. 2023)], we suggest a Phase IIb (proof of concept) clinical trial for fingolimod, with a cohort consisting of elderly people with mild cognitive impairment (MCI), to test whether oral medication with fingolimod (Gilenya) retards or reverses cognitive decline.

*Inclusion criteria*: The cohort should consist of male and female elderly patients aged 60–80 years with (according to NIA-AA guidelines; (Albert et al. 2011)) clinical amnestic MCI defined by an age, sex, and education-adjusted performance below -1.5 standard deviations (SD) on the delayed recall trial of the word-list episodic memory tests of the CERAD neuropsychological test battery (Fillenbaum et al. 2008), and showing a mini mental status evaluation (MMSE) score > 24. A biomarker assessment in CSF or blood should yield an Aβ42/40 ratio ≤ 0.08 and be positive for phospho-tau, to identify patients with prodromal AD (MCI due to Alzheimer’s disease). Alternatively, positron emission tomography (PET) measurement can be used to confirm brain amyloid pathology.

*Exclusion criteria:* Due to known fingolimod side effects, patients with known macula edema as well as coronary heart disease in all manifestations, arrhythmias, clinically relevant cardiac defects, heart insufficiency or resting bradycardia should be excluded. Likewise, due to immune-suppressive effects of fingolimod, patients with acute/chronic viral (e.g., hepatitis) or bacterial (e.g., tuberculosis) infection have to be excluded. Of note, patients who presented other clinically significant lesions on brain MRI, which could indicate a dementia diagnosis other than Alzheimer's disease should be excluded.

*Treatment*: In accordance with the well-established fingolimod doses that are FDA-approved for treatment of MS patients, we suggest oral daily medication with 0.25 or 0.5 mg fingolimod for 6–12 months.

*Primary efficacy endpoint*: Better cognitive performance assessed with the PACC5 (preclinical Alzheimer’s cognitive composite (Papp et al. 2017)) after 6−12 months treatment in fingolimod vs. control group.

*Key secondary endpoints*: For objective detection of FTY720 treatment-induced improvements in brain structure and metabolism, magnetic resonance imaging (MRI) should be used to test volume changes and signs of less atrophy in neocortex and hippocampus of fingolimod treated patients. Moreover, PET with radioactively labeled glucose (FDG) might be used to measure glucose turnover in the hippocampus and neocortex as a proxy for increased brain activity and synaptic function (Jack et al. 2016). These analyzes should be performed before and after the end of FTY720 treatment and changes that occur would be compared to placebo controls.

*Safety considerations*: Due to known cardiac and immuno-suppressive actions of fingolimod, patients need to undergo monitoring of cardiac status (ECG) upon first medication, and monthly blood counts to detect potential immunosuppressive side effects (e.g., severe lymphopenia) and increased liver enzyme abnormalities. Unchanged risk of myocardial infarction, bradycardia, hypertension and acute infections in fingolimod vs. control group needs to be monitored. Data regarding adverse effects in patients > 65 years are available from the former MS trials and need to be carefully reflected by the study design. For clinical trials investigating ozanimod and siponimod, less cardiac side effects are expected (compare previous section), whereas immuno-suppressive effects need to be monitored as in case of treatment with FTY720.

## Conclusions and outlook

One of the main objectives of AD research is to identify novel therapeutic approaches alleviating cognitive deficits. The recent discovery that treatment with the anti-inflammatory drug fingolimod, which mimics the molecular structure of endogenous sphingosine-1-phosphate that is naturally generated in mammalian cells, and that is already approved as a drug for human use in MS, counteracts memory impairment in five different mouse models of AD, prompts to investigate its potential in treating prodromal AD patients. This approach is supported by the stunning recent finding that mutations in key enzymes of the cellular ceramide/sphingosine pathway enhance the risk to develop sporadic AD (Baloni et al. 2022). The novelty of the approach to repurpose fingolimod for the treatment of AD lies in the anti-inflammatory actions of fingolimod that can be used as oral medication to halt or reverse synaptic deficits and the associated cognitive dysfunctions in AD patients. Given the meanwhile well-defined cascade of amyloid-β and tau induced neuro-inflammatory effects in AD that are mediated via activated microglia, reactive astrocytes, and lymphocytes infiltrating the AD-diseased brain, anti-inflammatory treatment of AD patients seems appropriate and promising to deliver one of the first disease modifying therapies against AD.

## Data Availability

This is a review article, and therefore does not contain experimental data that could be made available.
